# Supplementary material for: Ultrafast Comparison of Personal Genomes via Precomputed Genome Fingerprints
Source: Front Genet. 2017 Sep 26;8:136. doi: 10.3389/fgene.2017.00136 (PMC5623000; doi:10.3389/fgene.2017.00136)
Supplement: Supplementary file 1 [file Data_Sheet_1.DOCX]

Supplementary Material

Ultrafast comparison of personal genomes

Gustavo Glusman^*^, Denise E. Mauldin, Leroy E. Hood, Max Robinson

*** Correspondence:** Gustavo Glusman: Gustavo@SystemsBiology.org

# Supplementary Figures and Tables

## Supplementary Figures

**Supplementary Figure 1.** Technology effects on variants at short distances from each other. We computed the fraction of SNV pairs as a function of the distance (number of intervening nucleotides) between consecutive SNVs for various versions of the genome of the same individual (NA12878). There is a clear discrepancy between genomes sequenced by Complete Genomics (CGI) and by Illumina technologies in the frequency of SNVs at very short distances, particularly SNVs at adjacent positions or separated by one intervening nucleotide. Smaller differences are observed also up to 38 intervening nucleotides, consistent with the read length of CGI technology.


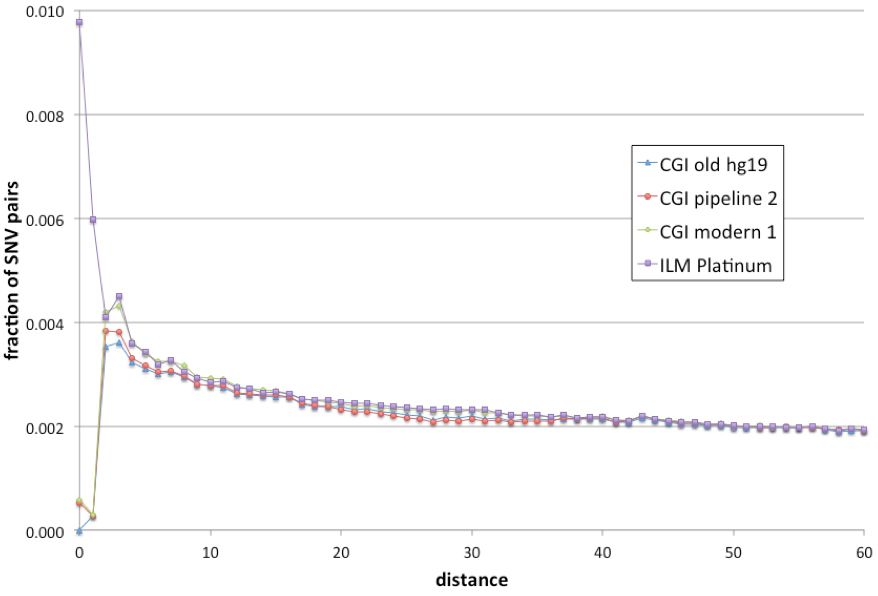


**Supplementary Figure 2.** Example of a raw fingerprint for NA12878, computed with *L*=120 and *C*=20, color-coded from lowest values (blue) through intermediate values (white) to highest values (red). The panels to the right and down represent the sums of values in rows (SNV pairs) and columns (distance modulo *L*), respectively.


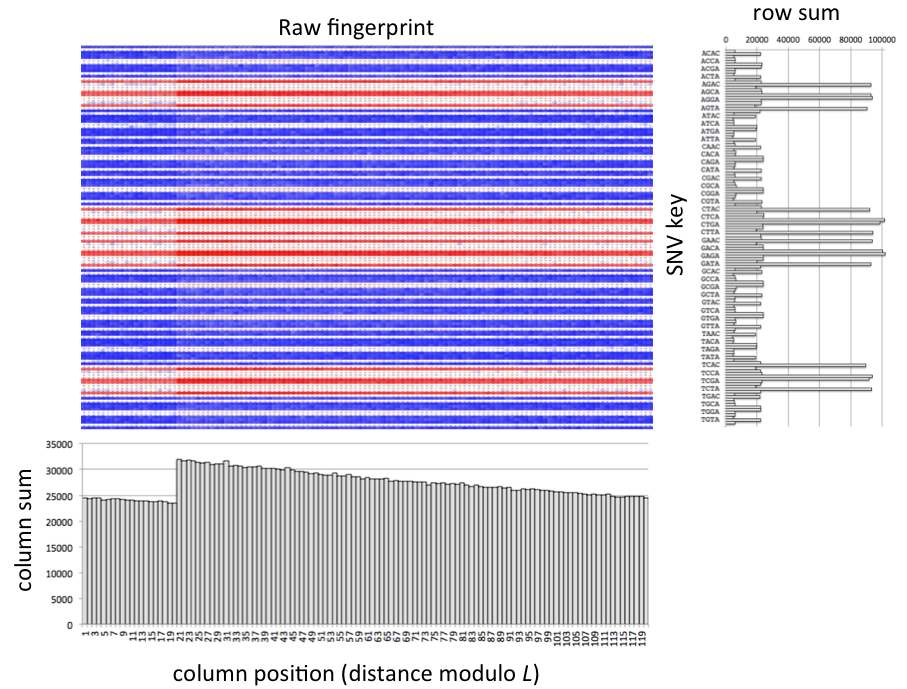


**Supplementary Figure 3.** Example of a normalized fingerprint for NA12878, computed with *L*=120 and *C*=20, color-coded from -4 (blue) through 0 (white) to 4 (red). The panels to the right and down represent the sums of values in rows (SNV pairs) and columns (distance modulo *L*), respectively.


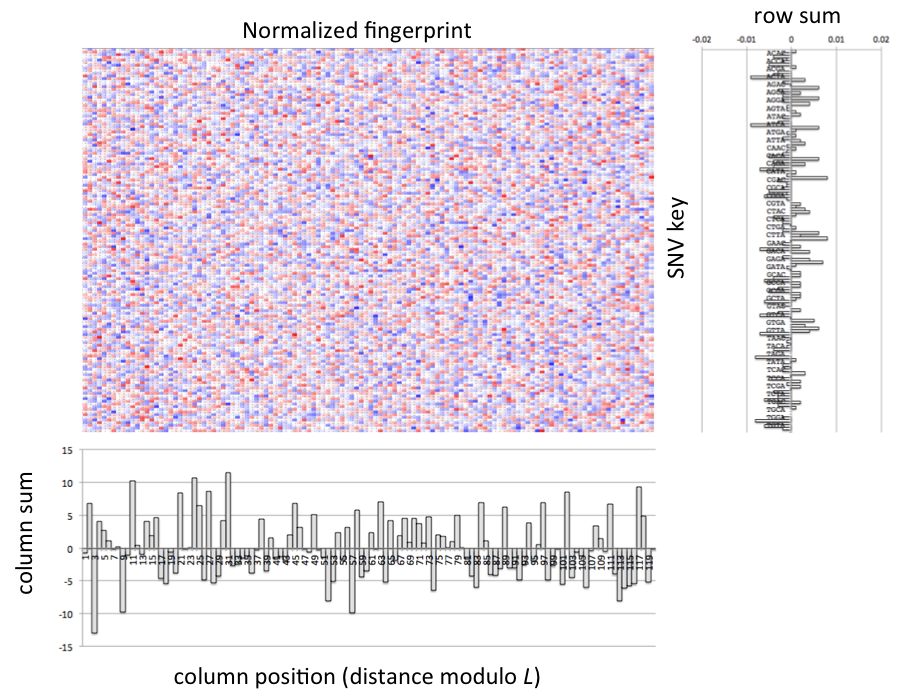


**Supplementary Figure 4.** Example of a fingerprint for NA12878, computed with *L*=120 and *C*=20 and normalized in reverse order (first rows, then columns), color-coded from -4 (blue) through 0 (white) to 4 (red). The panels to the right and down represent the sums of values in rows (SNV pairs) and columns (distance modulo *L*), respectively. This normalization order clearly fails to remove the internal structure of the raw fingerprint.


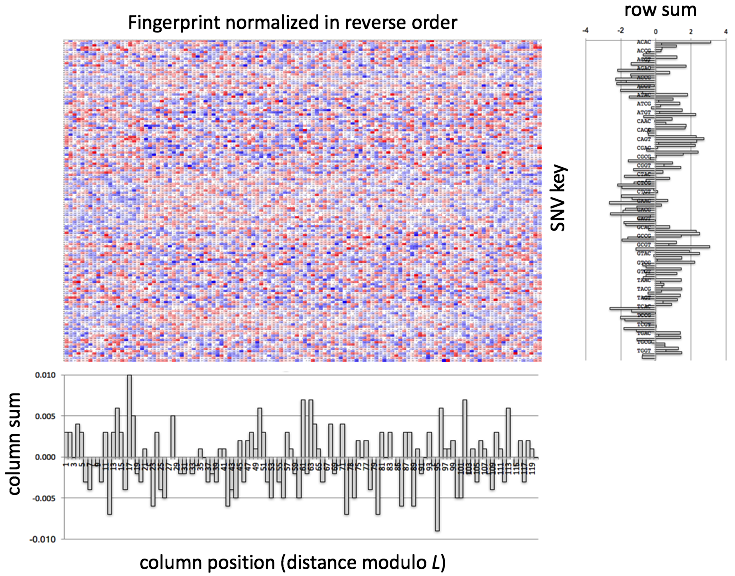


**Supplementary Figure 5.** Example of a population-adjusted fingerprint for NA12878, computed with *L*=120 and *C*=20 and using CEU as reference, color-coded from -4 (blue) through 0 (white) to 4 (red). The panels to the right and down represent the sums of values in rows (SNV pairs) and columns (distance modulo *L*), respectively.


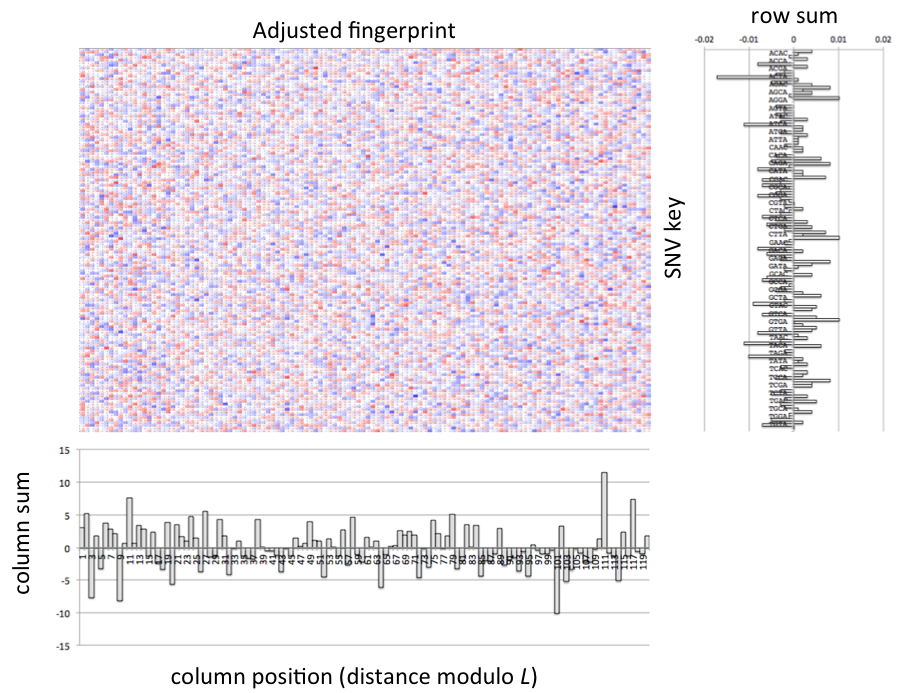


**Supplementary Figure 6.** Overview of method for computing binary fingerprints. Pairs of consecutive SNVs in the input file (upper right) are encoded into a table (upper left) by SNV key and by distance. A section of the table, informative about technology, is segregated (lower left). The rest of the table is folded using modulo 2 to generate a raw fingerprint (lower center), which is then binarized according to whether the value in the second column is larger than the value in the first column (lower right).


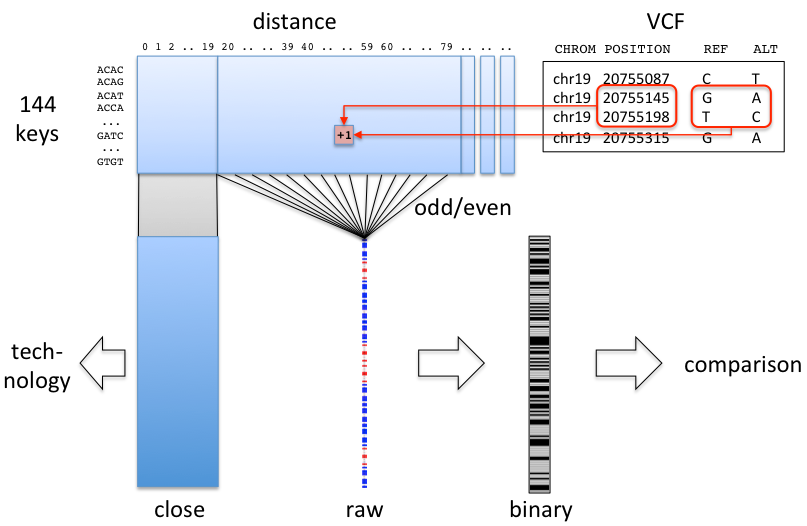


**Supplementary Figure 7.** Binary fingerprint match distributions. We compared binary fingerprints for 154 genomes from the Thousand Genomes dataset, each minimally processed and represented separately (single-VCF) or heavily post-processed and combined into a unified representation (multi-VCF). Solid lines represent all possible comparisons among different individuals; the dotted line represents the comparisons of both binary fingerprints for each of the 154 individuals. Black: comparison of genomes within the single-VCF set. Blue: comparison within the multi-VCF set. Red: cross-comparison of one genome from the single-VCF set and one from the multi-VCF set.

**
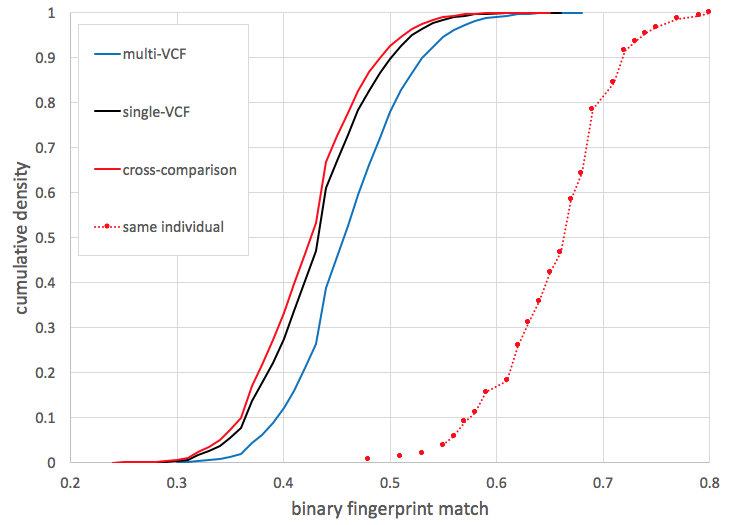
**

**Supplementary Figure 8.** Variation in fingerprint correlation. Each trace represents the standard deviation of correlations between fingerprints of individuals in each relatedness group, as a function of *L*. Details as in Figure 4.


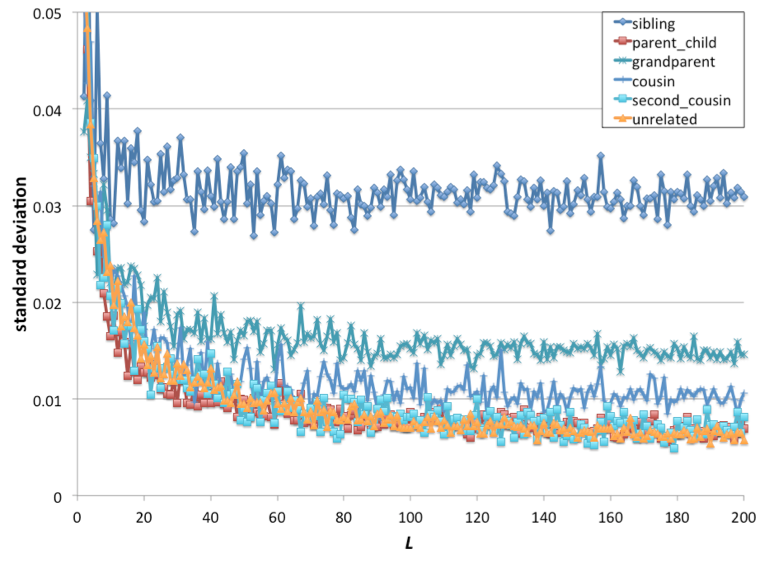


## Supplementary Tables

**Supplementary Table 1.** Likely related pairs from among the 2504 individuals in the 1000 Genomes data set, identified by correlation of population-adjusted fingerprints. The Relationship column is from Supplemental Table 2 reported by Gazal et al., Sci Rep 2015, 5:17453; these relationship assignments were inferred using RELPAIR. FS: full siblings. PO: parent/offspring. HS: half siblings. AV: avuncular. CO: cousins.

| **Genome 1** | **Genome 2** | **Correlation** | **Population 1** | **Population 2** | **Relationship** |
| --- | --- | --- | --- | --- | --- |
| HG03873 | HG03998 | 0.428 | ITU | STU |  |
| HG03733 | HG03899 | 0.415 | STU | STU | FS |
| HG02429 | HG02479 | 0.406 | ACB | ACB | FS |
| NA19331 | NA19334 | 0.361 | LWK | LWK | FS |
| NA20882 | NA20900 | 0.344 | GIH | GIH | PO |
| HG03750 | HG03754 | 0.330 | STU | STU | PO |
| NA20891 | NA20900 | 0.325 | GIH | GIH | PO |
| NA20320 | NA20321 | 0.316 | ASW | ASW | PO |
| NA20355 | NA20334 | 0.310 | ASW | ASW | PO |
| NA20359 | NA20362 | 0.304 | ASW | ASW | PO |
| NA20317 | NA20318 | 0.297 | ASW | ASW | PO |
| NA19904 | NA19913 | 0.295 | ASW | ASW | PO |
| NA21109 | NA21135 | 0.143 | GIH | GIH | HS |
| NA19027 | NA19042 | 0.142 | LWK | LWK | AV |
| HG03352 | HG03343 | 0.118 | ESN | ESN | CO |
| NA19625 | NA20274 | 0.110 | ASW | ASW | AV |
| HG02658 | HG02657 | 0.105 | PJL | PJL | CO |
| HG00116 | HG00120 | 0.103 | GBR | GBR | CO |
| HG00475 | HG00542 | 0.103 | CHS | CHS | AV |
| HG02691 | HG02690 | 0.098 | PJL | PJL | CO |
| HG02681 | HG02682 | 0.095 | PJL | PJL | CO |
| HG03228 | HG03229 | 0.092 | PJL | PJL | CO |
| HG03866 | HG03873 | 0.089 | ITU | ITU | CO |
| HG02699 | HG02700 | 0.086 | PJL | PJL | CO |
| NA19307 | NA19312 | 0.086 | LWK | LWK | CO |
| HG03301 | HG03372 | 0.085 | ESN | ESN | CO |
| NA19384 | NA19025 | 0.082 | LWK | LWK | CO |
| NA19355 | NA19434 | 0.081 | LWK | LWK | CO |
| HG00851 | HG00881 | 0.081 | CDX | CDX | CO |
| NA20864 | NA20891 | 0.078 | GIH | GIH | CO |
| HG03464 | HG03484 | 0.077 | MSL | MSL | CO |
| HG03896 | HG03692 | 0.077 | STU | STU | CO |
| HG04161 | HG04162 | 0.077 | BEB | BEB | CO |
| HG00238 | HG00240 | 0.076 | GBR | GBR | CO |
| HG03073 | HG03097 | 0.072 | MSL | MSL | CO |
| HG03428 | HG03457 | 0.072 | MSL | MSL | CO |
| NA19451 | NA19452 | 0.071 | LWK | LWK | CO |
| HG02356 | HG02379 | 0.071 | CDX | CDX | CO |
| HG02648 | HG02649 | 0.069 | PJL | PJL | CO |
| HG03478 | HG03484 | 0.068 | MSL | MSL | CO |
| HG03478 | HG03469 | 0.067 | MSL | MSL | CO |
| HG03955 | HG03898 | 0.067 | STU | STU | CO |
| NA12383 | NA11932 | 0.065 | CEU | CEU | CO |
| HG03866 | HG03998 | 0.064 | ITU | STU |  |
| HG01795 | HG02179 | 0.063 | CDX | CDX | CO |
| HG03352 | HG03366 | 0.063 | ESN | ESN | CO |
| HG00607 | HG00581 | 0.062 | CHS | CHS | CO |
| NA19657 | NA19786 | 0.062 | MXL | MXL | CO |
| HG00584 | HG00595 | 0.061 | CHS | CHS | CO |
| HG00112 | HG00123 | 0.060 | GBR | GBR | CO |
| HG02156 | HG02380 | 0.060 | CDX | CDX | CO |
| HG02353 | HG02379 | 0.059 | CDX | CDX | CO |
| HG03733 | HG03955 | 0.056 | STU | STU | CO |
| HG03464 | HG03478 | 0.054 | MSL | MSL | CO |
| NA19732 | NA19731 | 0.053 | MXL | MXL |  |
| NA19347 | NA19376 | 0.051 | LWK | LWK | CO |
| NA19728 | NA19731 | 0.051 | MXL | MXL |  |
| HG03899 | HG03898 | 0.049 | STU | STU | CO |
| NA19729 | NA19732 | 0.049 | MXL | MXL | CO |
| HG02272 | HG02271 | 0.048 | PEL | PEL | CO |
| NA19428 | NA19461 | 0.048 | LWK | LWK | CO |
| HG01468 | HG01465 | 0.047 | CLM | CLM | CO |
| HG02624 | HG02610 | 0.047 | GWD | GWD | CO |
| NA19317 | NA19376 | 0.047 | LWK | LWK | CO |
| NA19452 | NA19320 | 0.047 | LWK | LWK | CO |
| NA19661 | NA19752 | 0.047 | MXL | MXL | CO |
| HG03836 | HG03752 | 0.045 | STU | STU | CO |
| HG03899 | HG03955 | 0.045 | STU | STU | CO |
| HG04023 | HG04025 | 0.045 | ITU | ITU | CO |
| HG02685 | HG02684 | 0.043 | PJL | PJL | CO |
| HG02687 | HG03705 | 0.043 | PJL | PJL | CO |
| NA19350 | NA19397 | 0.043 | LWK | LWK | CO |
| HG04023 | HG04026 | 0.042 | ITU | ITU |  |
| NA19430 | NA19037 | 0.042 | LWK | LWK | CO |
| NA19729 | NA19731 | 0.042 | MXL | MXL |  |
| NA19728 | NA19729 | 0.041 | MXL | MXL | CO |
| HG03234 | HG03235 | 0.040 | PJL | PJL | CO |
| HG03380 | HG03391 | 0.040 | MSL | MSL | CO |
| HG03896 | HG03894 | 0.039 | STU | STU | CO |
| HG03991 | HG03898 | 0.039 | STU | STU | CO |
| NA19028 | NA19385 | 0.039 | LWK | LWK | CO |
| NA19375 | NA19376 | 0.039 | LWK | LWK | CO |
| NA20340 | NA20346 | 0.039 | ASW | ASW | CO |
| HG01354 | HG01489 | 0.038 | CLM | CLM | CO |
| HG02353 | HG02386 | 0.038 | CDX | CDX | CO |
| HG02654 | HG02655 | 0.038 | PJL | PJL | CO |
| HG03955 | HG03991 | 0.038 | STU | STU | CO |
| HG04025 | HG04056 | 0.037 | ITU | ITU |  |
| NA19346 | NA19395 | 0.037 | LWK | LWK | CO |
| HG03773 | HG04026 | 0.036 | ITU | ITU |  |
| NA19451 | NA19037 | 0.036 | LWK | LWK | CO |
| NA19834 | NA19920 | 0.036 | ASW | ASW | CO |
| NA20864 | NA20900 | 0.036 | GIH | GIH | CO |
| HG03121 | HG02943 | 0.035 | ESN | ESN | CO |
| HG01961 | HG02272 | 0.034 | PEL | PEL |  |
| HG03733 | HG03898 | 0.034 | STU | STU | CO |
| HG03741 | HG03740 | 0.034 | STU | STU | CO |
| HG03784 | HG03861 | 0.034 | ITU | ITU |  |
| NA19347 | NA19320 | 0.034 | LWK | LWK | CO |
| HG03772 | HG03786 | 0.034 | ITU | ITU |  |
| HG01259 | HG01275 | 0.033 | CLM | CLM | CO |
| HG03738 | HG03837 | 0.033 | STU | STU | CO |
| HG04023 | HG04063 | 0.033 | ITU | ITU |  |
| NA19728 | NA19732 | 0.033 | MXL | MXL |  |
| HG01801 | HG02380 | 0.032 | CDX | CDX | CO |
| HG02464 | HG02804 | 0.032 | GWD | GWD | CO |
| HG03861 | HG04056 | 0.032 | ITU | ITU |  |
| HG04017 | HG04096 | 0.032 | ITU | ITU |  |
| NA19451 | NA19320 | 0.032 | LWK | LWK | CO |
| HG02345 | HG01971 | 0.031 | PEL | PEL |  |
| HG03896 | HG03995 | 0.031 | STU | STU | CO |
| NA19360 | NA19434 | 0.031 | LWK | LWK | CO |
| HG01259 | HG01437 | 0.030 | CLM | CLM |  |
| HG02259 | HG02291 | 0.030 | PEL | PEL |  |
| HG03268 | HG03271 | 0.030 | ESN | ESN | CO |
| HG03733 | HG03991 | 0.030 | STU | STU | CO |
| HG03784 | HG04096 | 0.030 | ITU | ITU |  |
| HG04096 | HG03772 | 0.030 | ITU | ITU |  |
| NA20786 | NA20811 | 0.030 | TSI | TSI | CO |
| HG01893 | HG01566 | 0.029 | PEL | PEL |  |
| HG02272 | HG02291 | 0.029 | PEL | PEL |  |
| HG02694 | HG02690 | 0.029 | PJL | PJL | CO |
| HG03718 | HG04096 | 0.029 | ITU | ITU |  |
| HG03785 | HG04056 | 0.029 | ITU | ITU |  |
| HG03943 | HG04106 | 0.029 | STU | STU |  |
| HG04025 | HG04054 | 0.029 | ITU | ITU |  |
| NA12830 | NA12760 | 0.029 | CEU | CEU | CO |
| NA19375 | NA19436 | 0.029 | LWK | LWK | CO |
| HG01920 | HG02291 | 0.028 | PEL | PEL |  |
| HG03717 | HG03786 | 0.028 | ITU | ITU |  |
| HG03773 | HG04025 | 0.028 | ITU | ITU |  |
| HG04017 | HG04026 | 0.028 | ITU | ITU |  |
| HG04025 | HG04096 | 0.028 | ITU | ITU |  |
| HG04056 | HG04054 | 0.028 | ITU | ITU |  |
| NA19648 | NA19679 | 0.028 | MXL | MXL |  |
| NA19657 | NA19785 | 0.028 | MXL | MXL | CO |
| HG02610 | HG02642 | 0.028 | GWD | GWD | CO |
| HG00475 | HG00500 | 0.027 | CHS | CHS | CO |
| HG01920 | HG01961 | 0.027 | PEL | PEL |  |
| HG02265 | HG02291 | 0.027 | PEL | PEL |  |
| HG03557 | HG03556 | 0.027 | MSL | MSL | CO |
| HG03785 | HG04096 | 0.027 | ITU | ITU |  |
| HG03899 | HG03991 | 0.027 | STU | STU | CO |
| HG04096 | HG03786 | 0.027 | ITU | ITU |  |
| NA20770 | NA20531 | 0.027 | TSI | TSI | CO |
